# A comparison of modeling approaches when estimating tibial loading during running with different foot strike patterns

**DOI:** 10.7717/peerj.21328

**Published:** 2026-05-25

**Authors:** Sanghyuk Han, Matthew Ellison, Akbar Javadi, Dominic J. Farris, Hannah Margaret Rice

**Affiliations:** 1 Public Health and Sport Sciences, University of Exeter, Exeter, United Kingdom; 2 Department of Engineering, University of Exeter, Exeter, United Kingdom; 3 Department of Physical Performance, Norwegian School of Sport Sciences, Oslo, Norway

**Keywords:** Internal tibial loading, 2D Beam theory, 3D Finite element analysis, Statistical shape modeling, Foot strike pattern

## Abstract

**Background:**

Foot strike can alter internal tibial loading, a mechanical factor linked to tibial stress injury. Participant-specific models are essential for evaluating these loads, but detailed tibial geometry typically requires medical imaging. Open-source statistical shape modeling (SSM) enables geometric reconstruction of the tibia from external landmarks without imaging. However, the reconstruction accuracy achievable using only four anatomical markers commonly used in motion capture, relative to imaging-based reference geometries, is unknown. It also remains unclear whether simplified 2D beam theory and detailed 3D finite element analyses produce consistent internal tibial loading responses across altered running conditions. This study (1) quantified reconstruction errors using a 4-marker SSM configuration; and (2) evaluated the consistency of tibial loading estimates across modeling approaches under different foot strike conditions.

**Methods:**

An open-source anatomical dataset (*n* = 35) was used to assess reconstruction accuracy between 4- and 9-marker SSM configurations under varying principal component constraints, relative to MRI-based reference geometries, using the Jaccard index and surface error metrics. A 4-marker SSM approach generated tibial geometries for participants (*n* = 18) who completed running trials at 4.0 m ⋅ s^−1^ under habitual and imposed rearfoot and forefoot strike conditions. Muscular forces were estimated using static optimization. Tibial stress and strain were estimated using 2D beam theory and 3D finite element analysis (FEA) with identical loading inputs. Friedman tests with Wilcoxon *post hoc* tests and Bonferroni correction (*p*_corr_ < 0.05) were used for comparisons, and Spearman’s correlation assessed tibial length agreement.

**Results:**

The 4-marker configuration showed a 17.1% lower Jaccard index than the 9-marker configuration. Reconstructed tibial lengths correlated with marker-based measurements (Spearman’s *ρ* = 0.670, *p* = 0.002), with a mean absolute error of 13.8 mm and a 1.3% average length difference. During imposed forefoot striking, peak anterior tibial stress increased by 17.0% (2D) and 18.8% (3D) relative to habitual rearfoot striking. Participant-specific percentage changes were directionally consistent across methods, with a mean absolute between-method difference of 5.3%.

**Conclusion:**

Relative changes in internal tibial loading showed consistent directional trends between approaches across foot strike conditions, indicating that simplified 2D beam theory may provide a practical alternative for estimating within-participant or within-group changes in healthy runners when medical imaging is unavailable.

## Introduction

Lower limb overuse injuries are common in distance runners, and tibial stress injuries represent a significant proportion of these (Van Gent et al., 2007; Robertson & Wood, 2017). These injuries arise primarily from mechanical fatigue, characterized by repeated submaximal loading leading to cumulative microdamage in cortical bone (Burr et al., 1997; Zioupos, Wang & Currey, 1996; Edwards, 2018). When microdamage accumulates faster than bone remodeling can repair it, bone stress injuries can occur, resulting in prolonged rehabilitation and training disruptions (Carter & Caler, 1985; Warden, Davis & Fredericson, 2014). Running mechanics, including foot strike, influence external biomechanical loading measures that have been associated with the risk of tibial stress injury (Huang et al., 2019; Yong et al., 2018).

Previous studies have reported that foot strike patterns influence external forces and injury risk (Daoud et al., 2012; Zadpoor & Nikooyan, 2011). However, a topical review concluded that there is limited evidence that changing foot strike pattern reduces the risk of running-related injuries (Hamill & Gruber, 2017). Consistent with this, a systematic review found low evidence to suggest a relationship between foot strike pattern and running-related injuries (Burke et al., 2021). The transition from a rearfoot to a forefoot strike pattern has been associated with reductions in peak and average loading rates of the ground reaction force (GRF) (Boyer, Rooney & Derrick, 2014; Zadpoor & Nikooyan, 2011; Huang et al., 2019). Nonetheless, foot strike can change the loading environment of the tibia, and better understanding of this is warranted.

External GRF metrics have been widely used when assessing running foot strike patterns, yet they do not necessarily correlate strongly with internal tibial loading. Tibial loading refers broadly to the internal stress- and strain- experienced by the tibia, which can be estimated using different approaches. External proxy measures, including GRF metrics and peak tibial accelerations, have been shown to be unreliable indicators of internal tibial loading and may misrepresent changes associated with altered running conditions (Matijevich et al., 2019; Zandbergen et al., 2025; Van den Berghe et al., 2025). In our recent study (Han et al., 2025), we quantified internal tibial loading using musculoskeletal modeling and beam theory, comparing habitual and imposed foot strike patterns without incorporating participant-specific tibial geometry. We observed significantly greater tibial bending moments and cumulative-weighted tibial impulses when runners adopted an imposed forefoot strike compared to habitual or imposed rearfoot strikes. However, these analyses were conducted using a pre-determined estimate of bone geometry applied to all participants, rather than participant-specific modeling approaches used previously (Rice et al., 2020; Haider, Baggaley & Edwards, 2020; Bruce et al., 2022b).

Statistical shape modeling (SSM) enables reconstruction of participant-specific tibial geometry without the need for medical imaging, typically using external anatomical landmarks (Nolte et al., 2020; Bruce et al., 2022b; Keast, Bonacci & Fox, 2023). Recently, Keast, Bonacci & Fox (2023) developed an open-source statistical shape model of the tibia-fibula derived from computed tomography scans of 30 cadavers, demonstrating accurate geometric reconstructions. Previous studies have commonly used landmark configurations requiring nine or more anatomical points, limiting their clinical and laboratory applicability due to the complexity and time involved in accurately identifying and digitizing these points (Bruce et al., 2022b; Zhang et al., 2016). Bruce et al. (2022b) reported that reconstructions using nine palpable landmarks showed slightly reduced but practically acceptable accuracy compared to a 14-landmark approach. Further simplifying landmark configurations, Zhang et al. (2016) improved the reconstruction accuracy of lower limb geometries using only seven skin-mounted motion capture landmarks compared to traditional isotropic scaling methods. However, reconstructions based on skin-mounted landmarks inherently involve uncertainties due to soft tissue offsets and marker placement errors (Nolte et al., 2020; Bruce et al., 2022b). On the other hand, Nolte et al. (2020) demonstrated that SSM reconstructions using skin-mounted landmarks consistently provided superior accuracy relative to conventional linear scaling methods, supporting the feasibility of using simplified landmark sets when access to more detailed participant-specific landmark data is not available.

In biomechanical motion capture, four palpable anatomical landmarks on the shank (the medial and lateral femoral epicondyles and medial and lateral malleoli) are widely used as anatomical reference points for defining lower limb segments and are often used for length-based scaling of biomechanical models. It is therefore possible to reconstruct the tibia based solely on these four practical landmark-based markers. In SSM, reconstruction accuracy depends on how effectively external anatomical landmarks constrain the principal component analysis (PCA) shape space during optimization. Each landmark provides geometric information that limits the range of admissible shapes. When fewer landmarks are used, the reconstruction is constrained by less geometric information, increasing uncertainty in shape features that are not directly defined by the remaining landmarks, such as tibial width, curvature, and distal–proximal flaring. Consequently, multiple anatomically plausible geometries can satisfy the same reduced landmark configuration, resulting in greater reconstruction error. Previous studies have demonstrated that reducing the number of anatomical landmarks used for SSM reconstruction increases surface error and reduces geometric accuracy (Bruce et al., 2022b), and that reconstruction accuracy in landmark-based SSM is strongly influenced by the extent to which external landmarks constrain the shape space during optimization (Nolte et al., 2020). However, the accuracy of reconstruction of the tibial geometry based solely on these four practical landmarks has not yet been assessed.

Tibial length significantly influences reconstruction accuracy for SSM (Keast, Bonacci & Fox, 2023), thus it may be feasible to generate usable tibial geometries from four landmarks that in addition indicate segment length. Evaluation of the reconstruction accuracy of this minimal landmark set is essential for participant-specific tibial modeling in order to quantify the extent of errors that may be introduced. Furthermore, reconstruction accuracy is influenced by PCA constraints, where higher constraints (*e.g.*, ±3 standard deviations (SD)) permit greater anatomical variation, potentially enhancing participant-specific geometry, but increase the risk of anatomically unrealistic deviations, while lower SD constraints (*e.g.*, ±1 SD) minimize unrealistic deviations but may inadequately capture individual anatomical variability.

In addition to geometric reconstruction accuracy, it remains important to determine whether different computational modeling approaches yield consistent trends in internal tibial loading across running conditions. Computational approaches for estimating tibial stress vary in complexity, ranging from simplified beam theory estimates to detailed participant-specific finite element analysis. Beam theory models approximate the tibia as a simplified geometric cross-section and are computationally efficient, allowing quantification of changes in internal bone loading within participants across conditions (Rice et al., 2019; Meardon & Derrick, 2014; Rice et al., 2020). However, these simplified models tend to underestimate axial stresses and overestimate bending stresses, limiting their validity for predicting internal bone loading, particularly between groups (Brassey et al., 2013).

In contrast, finite element analysis provides more realistic estimations by incorporating complex geometry, heterogeneous material properties, and relevant boundary conditions. This approach can reveal detailed, site-specific strain distributions and identify localized injury risks that would be overlooked by simplified beam theory models (Brassey et al., 2013; Baggaley et al., 2024; Baggaley, Derrick & Edwards, 2023). However, finite element analysis typically demands significant computational resources and detailed participant-specific geometry, which can limit the sample sizes that can be assessed. Combining finite element analysis with SSM may provide a compromise between these approaches, by providing quasi participant-specific geometry which is an improvement on non-specific approaches, yet remains computationally viable for assessing mechanistic or applied research questions, while avoiding the requirement for medical imaging.

This study aimed to quantify reconstruction errors in participant-specific tibial geometries generated from a simplified 4-marker configuration compared to an established 9-marker configuration, to evaluate the feasibility of this approach in the absence of participant-specific imaging. In addition, tibial loading (stress and strain) estimates under different foot strike patterns were evaluated using two computational modeling approaches—a simplified two-dimensional (2D) beam theory and a three-dimensional (3D) finite element analysis—based on SSM-reconstructed tibial geometry. It was hypothesized that the simplified 4-marker configuration would yield greater reconstruction errors than the 9-marker configuration, with reconstruction accuracy decreasing as PCA constraints increased (±1 to ±3 SD). It was further hypothesized that both modeling approaches would capture similar directional changes in internal tibial loading across foot strike patterns, including increased loading during an imposed forefoot strike. As a secondary, exploratory analysis, sex-based comparisons were included to assess whether the proposed modeling framework preserves known variability in tibial geometry and internal loading.

## Methods

### Datasets

This study utilized two independent datasets from distinct participant samples. First, an open-source magnetic resonance imaging (MRI)-based anatomical dataset (Nolte et al., 2016), which included nine anatomical landmarks positioned on the tibia–fibula complex, was used to evaluate tibial geometry reconstruction accuracy. In this context, MRI-derived tibial geometries served as the sole reference standard for assessing reconstruction error.

Second, a running biomechanics dataset of synchronized kinematic and kinetic data collected during overground running (Han et al., 2025) was used to estimate internal tibial loading across different foot strike conditions. Running kinematics and kinetics were applied only to statistical shape model–reconstructed geometries derived from the motion-capture dataset.

The statistical shape model itself was previously developed from computed tomography (CT) data (Keast, Bonacci & Fox, 2023) and was not developed from the MRI dataset used here.

#### MRI-based anatomical dataset

Reconstruction accuracy was assessed using an independent MRI-based anatomical validation dataset from Nolte et al. (2016), which included 35 healthy adults (13 females, 22 males; age: 23–70 years; height: 155–193 cm; body mass: 45–108 kg).

#### Running biomechanics dataset

The running-based experimental dataset comprised 19 healthy recreational runners (10 females, nine males; mean age: 34.0 ± 4.8 years; height: 166.4 ± 6.3 cm; body mass: 61.6 ± 6.6 kg), originally recruited for a previous study in which participant-specific tibial geometry was not available (Han et al., 2025). Although foot strike can be quantified on a continuum rather than as strictly discrete patterns (Van den Berghe et al., 2022; Shorten & Pisciotta, 2017), participants were included if they could be categorised as habitual rearfoot strikers. All participants habitually used a rearfoot strike pattern, confirmed by the foot strike angle at initial contact (14.3 ± 4.3^∘^), which was defined as the angle between the foot and the ground in the sagittal plane. A rearfoot strike was classified as an angle greater than 8°, based on established criteria (Altman & Davis, 2012). Exclusion criteria included any lower limb musculoskeletal injury within the past year or habitual use of a non-rearfoot strike pattern. All procedures were approved by the Korea Institute of Sport Science Institutional Review Board (IRB No. KISS-1907-018-01). Written informed consent was obtained from all participants prior to data collection.

### Experimental protocol for running data

Participants ran in their own habitual running shoes; minimalist footwear was not permitted, and all participants were provided with tight-fitting laboratory clothing. Fifty retro-reflective markers were placed to track anatomical frames, with marker coordinates recorded at 200 Hz using 18 infrared cameras (Oqus 7+; Qualisys, Gothenburg, Sweden). Ground reaction forces were collected at 2000 Hz using a force plate (9287BA; Kistler, Winterthur, Switzerland), synchronized with the motion capture system. After a self-directed warm-up, participants ran overground at 4.0 m ⋅ s^−1^ along a 10 m wooden indoor runway, ensuring full right foot contact on the force plate, with running speed (±5%) monitored *via* timing gates (Witty; Microgate, Bolanzo, Italy). Following five successful habitual trials with no foot strike instruction, participants were instructed to adopt either an imposed rearfoot or forefoot strike in a randomized order. Each imposed condition was preceded by five familiarization trials, with additional trials permitted if required to ensure reliable adoption of the instructed pattern. Five successful trials were then recorded per condition. Foot strike patterns were monitored visually by an investigator, with motion capture data reviewed and additional verbal feedback provided when necessary.

### Data processing for running data

Marker coordinates and force data were filtered using a fourth-order zero-lag Butterworth filter with cutoff frequencies of 10 Hz and 20 Hz for kinematic and kinetic data, respectively (Bisseling & Hof, 2006). Kinematic and kinetic data were analyzed in Visual3D (V6; HAS-Motion, Burnaby, Canada) during the stance phase, defined as the time period when the filtered vertical GRF exceeded 10 N. Data were time-normalized to 101 points (0%–100% stance).

Static trials were used to determine bilateral joint centers (ankle, knee, hip). Joint angles were computed using the XYZ Cardan sequence, with each joint modeled with 6 degrees of freedom. Net joint moments followed the right-hand rule. Joint moments were expressed in the shank segment coordinate system, with ankle dorsiflexion defined as the positive sagittal-plane rotation, consistent with ISB reporting recommendations (Derrick et al., 2020). Joint reaction forces were calculated *via* inverse dynamics using a customized MATLAB script (R2021a; MathWorks, Natick, MA, USA), incorporating participant-specific anthropometric parameters (Shan & Bohn, 2003).

### Statistical shape model

The SSM used for tibial reconstruction was developed by Keast, Bonacci & Fox (2023), based on CT scans of 30 cadaveric tibia-fibula complexes. The scanned individuals had a mean age of 28.7 ± 6.7 years, height of 176.1 ± 11.6 cm, and body mass of 70.2 ± 11.4 kg. Inclusion was limited to individuals with a history of regular participation in impact-based physical activities (*e.g.*, team sports, dance, recreational running or walking). The original SSM framework incorporated a 9-marker anatomical configuration defined on the tibia–fibula complex, which served as the reference landmark set in the present study. The CT scans were used exclusively in the original development of the SSM and were not used in the present study. PCA constraints define the allowable shape variation from the mean tibial geometry, where higher SD limits permit greater anatomical variation but increase the risk of unrealistic deviations.

### Tibial geometry reconstruction

Participant-specific tibial geometry was reconstructed by fitting anatomical landmark coordinates obtained from the MRI-based anatomical dataset to the statistical shape model through constrained optimization of principal component (PC) scores. PC scores were adjusted to minimize the distance between reconstructed and observed landmark locations, while being restricted to predefined bounds corresponding to the PCA constraints (*e.g.*, ±1, ±2, or ±3 SD). The resulting constrained PC scores were then used to generate the final tibial geometry, using both a 4-marker and the established 9-marker approach to allow quantification of the reconstruction errors. The reduced 4-marker configuration included the medial femoral condyle, lateral femoral condyle, medial malleolus, and lateral malleolus. These landmarks are commonly employed in biomechanical gait analyses due to their ease of palpation and role in defining the shank local coordinate system. A sensitivity analysis was conducted to evaluate how reconstruction accuracy was affected by varying PCA constraints (±1, ±2, and ±3 SD). The 9-marker configuration was held constant at ±3 SD to serve as a reference configuration consistent with prior implementations of this SSM (Keast, Bonacci & Fox, 2023). Sensitivity analysis of PCA constraints was conducted only for the simplified 4-marker configuration, as reduced landmark information may increase reconstruction uncertainty.

For the reconstruction accuracy assessment, landmark coordinates were taken from the MRI-based dataset (Nolte et al., 2016) and fitted using both the 4-marker and 9-marker configurations. Participant-specific tibial surfaces were reconstructed using the CT-derived statistical shape model and subsequently compared against the MRI-based reference surfaces from the validation dataset provided by Nolte et al. (2016). Reconstruction accuracy for the 4-marker set (±1, ±2, ±3 SD) was assessed by calculating volumetric overlap between reconstructed and reference geometries using the Jaccard index (values closer to 1 indicating higher overlap), and by evaluating mean and maximum surface errors. Surface error was calculated as the absolute Euclidean distance between corresponding surface nodes of the reconstructed and MRI-based reference geometries; therefore, mean and maximum surface errors represent absolute distances rather than signed values. Both the 4-marker and 9-marker reconstructions were evaluated by direct comparison with these MRI-based reference geometries. The 4-marker set was compared against an established 9-marker set (±3 SD), consisting of landmarks identified through palpation as previously described by Bruce et al. (2022b): tibial tuberosity, medial condyle, lateral condyle, medial malleolus, lateral malleolus, lateral aspect of the fibular head, and the anterior border of the tibia at 25%, 50%, and 75% of the distance from the medial condyle to the medial malleolus. The optimal PCA constraint for the 4-marker set was selected based on reconstruction accuracy metrics (Jaccard index, mean and maximum surface errors). Due to previously reported inaccuracies associated with fibular reconstructions even using the 9-marker set (Keast, Bonacci & Fox, 2023), the fibula was excluded from further analysis.

Subsequently, trabecular bone geometries were estimated from reconstructed cortical surfaces using a regression-based statistical prediction model provided by Keast, Bonacci & Fox (2023). This model predicts internal trabecular structures based on cortical surface geometry parameters, resulting in separate cortical and trabecular tibial geometries for each participant.

To evaluate the consistency between SSM-derived tibial length estimates and marker-based tibial length measures commonly used in motion capture studies, tibial lengths obtained from the participant-specific 3D reconstructions were compared against lengths measured from external motion capture markers.

**Figure 1:**
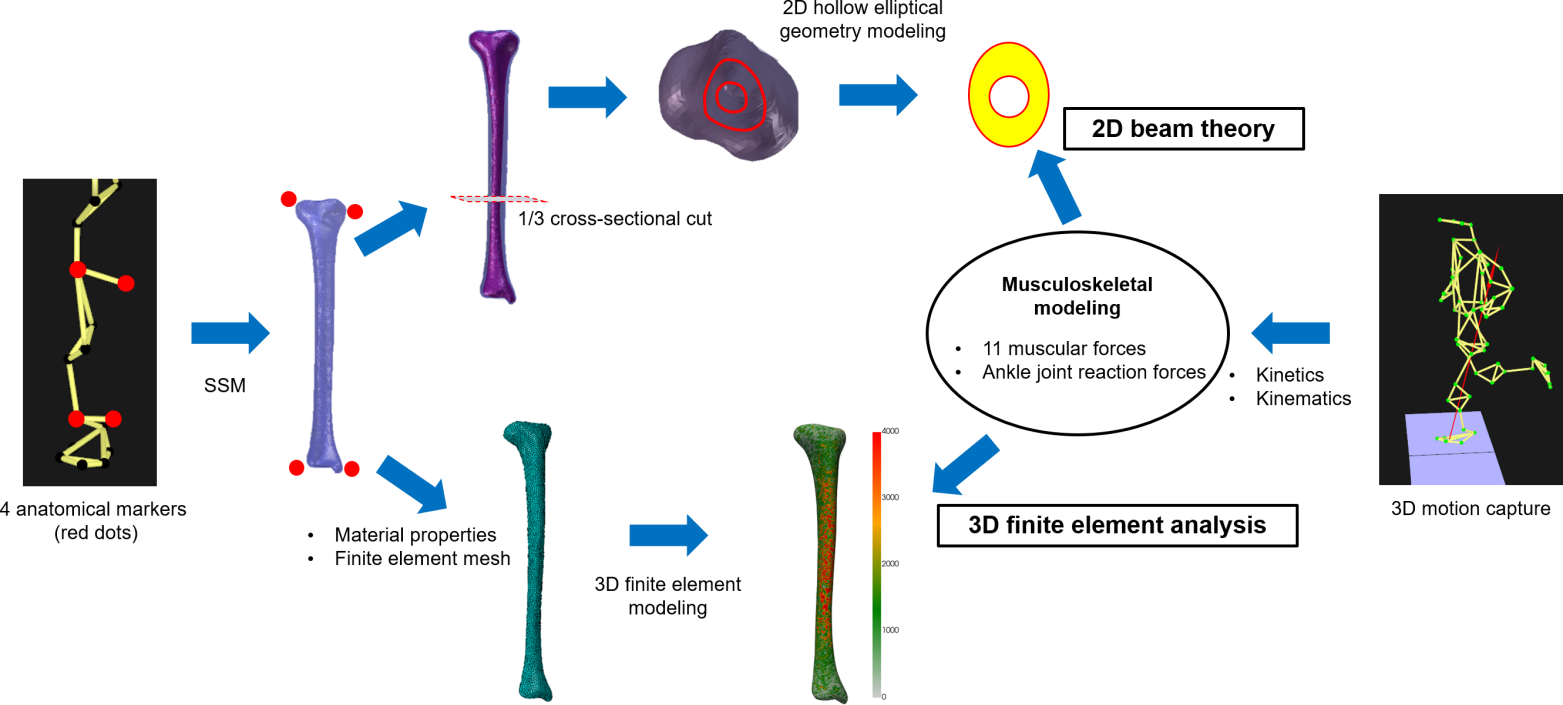
Schematic workflow for estimating tibial geometry using statistical shape modeling (SSM) and estimating tibial loading during running *via* 2D beam theory and 3D finite element analysis. The SSM was developed from CT-based medical imaging data (Keast, Bonacci & Fox, 2023).

Cross-sectional areas (CSA) for simplified 2D beam theory analysis were calculated at the distal third of the reconstructed tibial surfaces, a region commonly assessed for tibial loading, using Geomagic Design X software (2020.0; 3D Systems Inc., Rock Hill, SC, USA). The CSA was approximated as a hollow ellipse defined by maximal anteroposterior and mediolateral dimensions. Figure 1 provides a schematic overview of the methodological workflow.

### Musculoskeletal modeling

Muscular forces were estimated through static optimization, using the fmincon function in MATLAB which minimized the sum of cubed muscle stresses (Erdemir et al., 2007). 11 muscles spanning the tibia were included, and muscular parameters including physiological cross-sectional areas, muscle–tendon coordinates, and moment arms were obtained from the Hamner model (Hamner, Seth & Delp, 2010), consistent with the methods described in detail by Han et al. (2025). The resulting muscular force estimates were used as input for both the 2D beam theory and the 3D finite element analysis to quantify internal tibial loading under different foot strike conditions.

### 2D beam theory

The normal stress acting on the anterior and posterior aspects of the tibia was computed as the sum of axial stress and bending stress, consistent with methods detailed by Rice et al. (2019). Briefly, the axial stress was obtained by dividing the resultant axial force by the participant-specific CSA, and anterior-posterior normal stresses were calculated by adding or subtracting the bending stress component, determined using the bending moment about the medial-lateral axis and the second moment of area of the hollow elliptical cross-section. The bending moments used in this calculation were obtained as described by Han et al. (2025). By incorporating both axial and bending stress components, the anterior and posterior normal stresses were estimated for each participant under running conditions.

### 3D Finite element analysis

Finite element analysis was conducted using Abaqus CAE and the Abaqus Python Scripting Interface (version 2025; Dassault Systèmes Simulia Corp., Providence, RI, USA). Four mesh sizes (5.5 mm, 3.6 mm, 2.7 mm, and 2.0 mm) were evaluated in a mesh sensitivity analysis using 10-node quadratic tetrahedral elements. Mesh convergence was defined as percentage changes of less than 5% between successive refinements following established practices (Anderson, Ellis & Weiss, 2007; Henninger et al., 2010) for the following key variables: 95th percentile pressure-modified von Mises strain; peak principal tensile stress; peak principal compressive stress. Based on this analysis, a mesh size of 2.7 mm (161,843–261,857 elements from the shortest to the longest participant-specific tibia) was selected, similar to previous studies (Bruce et al., 2022a; Chen et al., 2016). Cortical and trabecular bones were modeled as isotropic materials with Young’s modulus of 17 GPa and 1 GPa, respectively, and a Poisson’s ratio of 0.3 (Edwards et al., 2009). Participant-specific bone density was not incorporated; instead, homogeneous material properties were applied consistently across all models.

Boundary conditions included a fully fixed proximal tibial plateau. The peak ankle joint contact forces (AJCF) were applied at a distal reference point. These forces, estimated along the axial, anterior–posterior (AP), and medial–lateral (ML) axes, were coupled to the distal tibial articular surface *via* a coupling constraint, ensuring uniform load distribution across the entire distal surface (Edwards et al., 2009; Chen et al., 2016). Since approximately 10% of the ankle joint force is typically transferred through the fibula—which was not included in our tibial-only model—the resultant ankle joint contact force applied to the tibia was scaled by 0.9 (Sasimontonkul, Bay & Pavol, 2007):

(1) \begin{eqnarray*}{F}_{AJCF}=0.9\cdot \left( R{F}_{\text{axial, AP, ML}}+\sum _{i=1}^{11}{f}_{i(\text{axial, AP, ML})} \right) \end{eqnarray*}

where *RF*_axial, AP, ML_ represents the ankle joint reaction force components, and (*f*_*i*(axial, AP, ML)_) represents the individual muscular forces in their respective directions. Both sets of forces were expressed along the axial, AP, and ML axes, defined relative to the longitudinal axis of the shank segment (Supplementary Material 1).

Pressure-modified von Mises equivalent strains were calculated based on the formulations by De Vree, Brekelmans & van Gils (1995) and Haider et al. (2021). Strained volume was defined as the volume of elements exceeding a threshold strain of 3000 *μɛ*, which has been associated with an increased risk of fatigue-induced damage (Haider et al., 2021). Finite element analyses were performed using loading conditions at the time of peak AJCF. From these analyses, the 95th percentile pressure-modified von Mises strain, peak principal tensile stress, and peak principal compressive stress were extracted for comparison across foot strike conditions.

### Statistical analysis

All statistical analyses were performed using IBM SPSS Statistics (version 26; IBM, Chicago, IL, USA). Given the within-participant repeated-measures design and the limited sample size, rank-based non-parametric statistical analyses were employed throughout, as these methods do not rely on distributional assumptions. Normality of all outcome variables was assessed using the Shapiro–Wilk test (*p* < 0.05) as a supplementary check.

Reconstruction accuracy for participant-specific tibial geometries was evaluated using the Jaccard index, mean surface error, and maximum surface error. The Jaccard index quantifies the degree of surface overlap between the SSM-reconstructed tibial geometry and the MRI-based tibial geometry, with a value of 1 indicating perfect overlap. Four marker configurations were evaluated: (1) 9-marker set (±3 SD); (2) 4-marker set (±3 SD); (3) 4-marker set (±2 SD); and (4) 4-marker set (±1 SD). Marker configuration was treated as a within-participant factor, as all configurations were evaluated for each participant. Differences across marker configurations were assessed using the Friedman test. *Post hoc* pairwise comparisons were conducted using Wilcoxon signed-rank tests with Bonferroni correction, where the significance threshold was determined by dividing 0.05 by the number of pairwise comparisons (*p*_corr_ < 0.05).

The geometric accuracy of reconstructed tibial lengths was evaluated by comparing tibial lengths obtained from SSM-based 3D reconstructions, generated using four motion capture markers, against tibial lengths directly measured between the corresponding four markers during the static calibration trial. Agreement between methods was assessed using Spearman’s rank correlation, mean absolute error, and a Wilcoxon signed-rank test (*p* < 0.05).

Foot strike condition effects on tibial loading-related variables were assessed separately for 2D beam theory and 3D finite element analysis. For 2D beam theory, peak anterior tensile stress and peak posterior compressive stress were analyzed. For 3D finite element analysis, the variables included 95th percentile pressure-modified von Mises strain, strained volume (elements exceeding 3000 *μɛ*), peak principal tensile stress, and peak principal compressive stress.

All variables were compared across foot strike conditions (habitual rearfoot strike, imposed rearfoot strike, and imposed forefoot strike) using the Friedman test. *Post hoc* pairwise comparisons between conditions were performed using Wilcoxon signed-rank tests, with Bonferroni correction applied for three comparisons (*p*_corr_ < 0.05). Effect sizes for Friedman tests were reported using Kendall’s W coefficient. Sex-based differences in CSA at the distal third of the tibia and peak anterior tibial stress (estimated using 2D beam theory) across foot strike conditions were assessed through exploratory descriptive statistics without formal statistical testing as a secondary analysis to demonstrate the potential applicability of the 2D beam theory approach.

## Results

One participant with a height of 151.8 cm was excluded from the 2D beam theory and 3D finite element analyses, as their height fell below the lower limit of the ±2 standard deviations range (152.8–199.3 cm) from the cadaver population used to develop the SSM (Keast, Bonacci & Fox, 2023).

Foot strike patterns for the participants in this study were previously reported by our group (Han et al., 2025). Foot strike angles (mean ± SD) were 14.3 ± 4.3^∘^ for habitual rearfoot strike, 29.0 ± 5.7^∘^ for imposed rearfoot strike, and −4.4 ± 4.7^∘^ for imposed forefoot strike. The corresponding 95% confidence intervals were 12.2–16.4^∘^, 26.3–31.8^∘^, and −6.7–−2.2^∘^, respectively, where a positive angle indicates the toes raised relative to the heel at the time of contact (Altman & Davis, 2012).

### 4-marker set SSM accuracy

Marker configuration influenced reconstruction accuracy metrics (*p* < 0.001, Table 1). Compared to the 9-marker set (±3 SD), all 4-marker configurations demonstrated lower Jaccard index values, with mean reductions of 14.6% for the ±1 SD configuration, 17.1% for the ±2 SD configuration, and 24.0% for the ±3 SD configuration (all *p*_corr_ < 0.05). Within the 4-marker configurations, Jaccard index values were higher for the ±1 SD and ±2 SD configurations compared to the ±3 SD configuration (both *p*_corr_ < 0.05), while no meaningful difference was observed between the ±1 SD and ±2 SD configurations.

**Table 1:** Reconstruction accuracy metrics for each marker configuration (mean (SD)).

	**9-marker set**	**4-marker set**	**4-marker set**	**4-marker set**	**Effect size**	***Post-hoc***
	**±3SD**	**±3SD**	**±2SD**	**±1SD**	**(Kendall’s W)**	
Jaccard index	0.64	0.49	0.53	0.55	0.517	A, B, C, D, E
	(0.05)	(0.10)	(0.09)	(0.10)		
Mean surface error (mm)	6.49	10.05	8.79	8.70	0.476	A, B, C, D, E
	(2.22)	(2.28)	(2.04)	(2.65)		
Max surface error (mm)	13.11	19.43	16.68	16.38	0.485	A, B, C, D, E
	(3.82)	(4.27)	(3.79)	(3.91)		

**Notes.**

Effect sizes are reported as Kendall’s *W* for Friedman tests. Significant *post-hoc* pairwise differences (*p*_corr_ < 0.05) are indicated as follows: A = 9-marker *vs* 4-marker ±3SD; B = 9-marker *vs* 4-marker ±2SD; C = 9-marker *vs* 4-marker ±1SD; D = 4-marker ±3SD *vs* 4-marker ±2SD; E = 4-marker ±3SD *vs* 4-marker ±1SD; F = 4-marker ±2SD *vs* 4-marker ±1SD.

Mean surface error differed across marker configurations (*p* < 0.001, Table 1). Compared to the 9-marker set, mean surface error increased by 34.1%, 35.5%, and 54.9% for the ±1 SD, ±2 SD, and ±3 SD configurations, respectively (all *p*_corr_ < 0.05). Within the 4-marker sets, the ±3 SD configuration resulted in larger mean surface errors than both the ±1 SD and ±2 SD configurations (both *p*_corr_ < 0.05), whereas no difference was observed between the ±1 SD and ±2 SD configurations.

A similar pattern was observed for maximum surface error (Table 1, *p* < 0.001). All 4-marker configurations exhibited larger maximum surface errors than the 9-marker set (all *p*_corr_ < 0.05), with the ±3 SD configuration showing the largest errors. Within the 4-marker configurations, maximum surface error was greater for the ±3 SD configuration compared to the ±1 SD and ±2 SD configurations (both *p*_corr_ < 0.05), while no difference was observed between the ±1 SD and ±2 SD configurations.

### Participant-specific model

The 4-marker set with ±2 SD limits was selected for generation of participant-specific tibial geometries based on reconstruction accuracy results. Tibial lengths derived from these SSM-based reconstructions demonstrated a significant positive correlation with lengths directly measured between external motion capture markers (Spearman’s *ρ* = 0.670, *p* = 0.002; Table 2). The mean absolute error between methods was 13.8 mm, and a Wilcoxon signed-rank test indicated no significant difference between marker-based and SSM-based tibial length estimates (*p* = 0.248).

**Table 2:** Descriptive statistics for tibial length and distal 1/3 tibial cross-sectional properties calculated using a hollow elliptical model (cross-sectional area (CSA); second moments of area (*I*_*ML*_: medial-lateral axis; *I*_*AP*_: anterior-posterior axis)). Tibial length was measured using Marker-based (3D motion capture) and SSM-based (reconstructed model) methods.

**Variables**	**Mean (SD)**
**Tibial length**	
Marker-based (mm)	398.2 (14.0)
SSM-based (mm)	403.6 (24.7)
**1/3 distal tibia**	
CSA (mm^2^)	393.1 (65.4)
*I*_*ML*_ (mm^4^)	23,607.5 (8,368.2)
*I*_*AP*_ (mm^4^)	18,894.2 (6,251.5)

Cross-sectional properties (CSA and second moments of area (*I*_*ML*_: medial-lateral axis; *I*_*AP*_: anterior-posterior axis)) at the distal 1/3 tibial location were calculated using a hollow elliptical model and are summarized in Table 2.

### 2D beam theory

**Figure 2:**
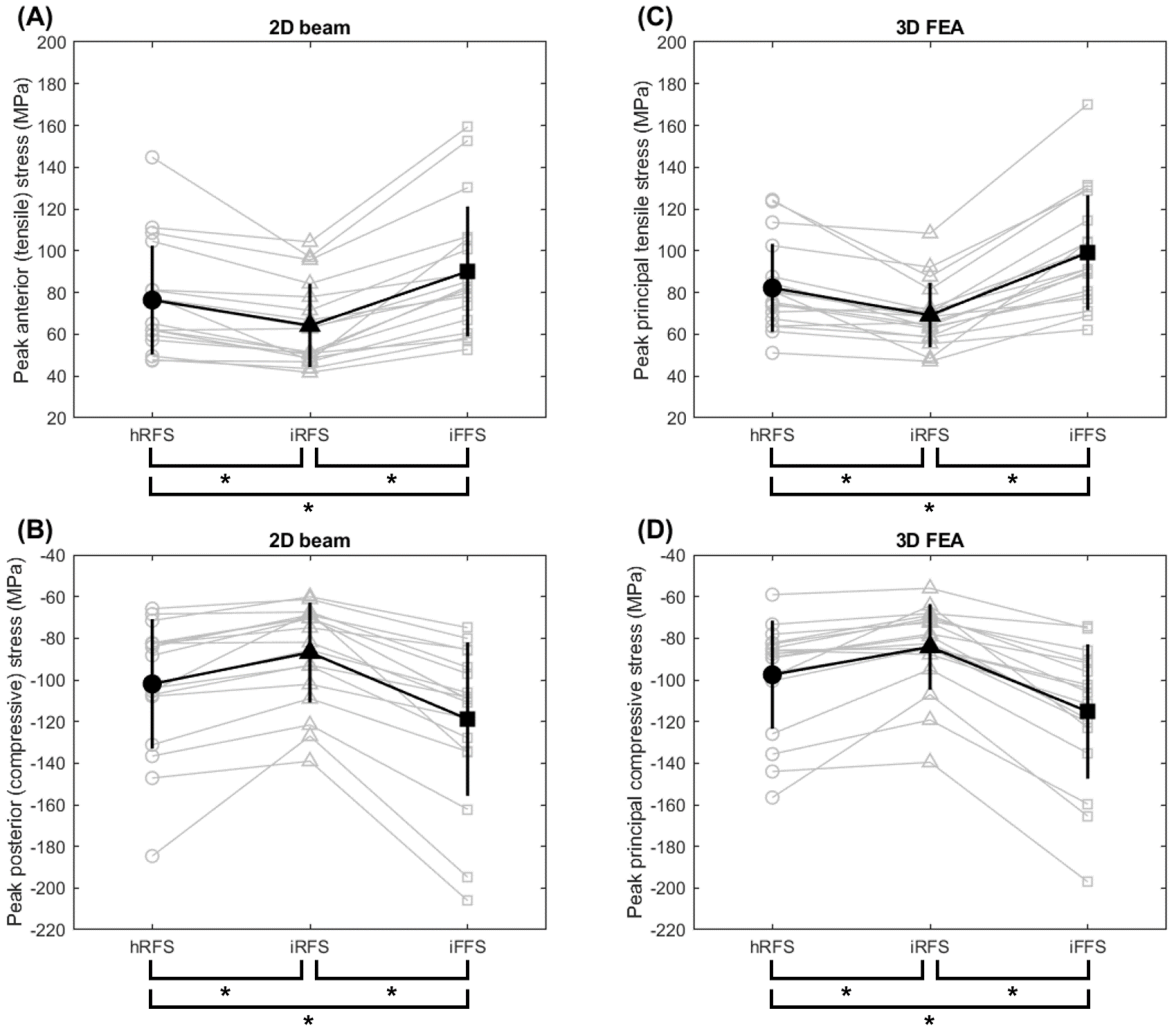
Peak tibial stresses estimated using two modeling approaches across foot strike conditions. Peak anterior (A) and posterior (B) stresses were estimated using the 2D beam theory, respectively. Peak principal tensile (C) and compressive (D) stresses estimated using 3D finite element analysis (FEA), respectively. Individual participant values are shown with condition-specific markers and connected by light gray lines to illustrate within-participant trends. Black filled markers and vertical error bars indicate the mean ± SD. hRFS, habitual rearfoot strike; iRFS, imposed rearfoot strike; iFFS, imposed forefoot strike. ∗ indicates a significant *post-hoc* difference between the connected conditions (*p*_corr_ < 0.05).

Figures 2A and 2B illustrate peak anterior (tensile) and posterior (compressive) tibial stresses estimated using 2D beam theory across foot strike conditions. Statistical analyses were performed for anterior stress only, as anterior and posterior stress measures exhibited highly similar condition-dependent patterns, consistent with previous findings (Han et al., 2025).

#### Peak anterior tensile stress

There was a main effect of foot strike pattern on peak anterior tensile stress at the distal third of the tibia (*p* < 0.001, Table 3). Compared to a habitual rearfoot strike, an imposed forefoot strike increased peak anterior tensile stress by 17.0% (*p*_corr_ < 0.05), and by 39.8% compared to an imposed rearfoot strike (*p*_corr_ < 0.05). Additionally, peak anterior tensile stress was lower by 16.3% with an imposed rearfoot strike compared to a habitual rearfoot strike (*p*_corr_ < 0.05).

**Table 3:** Tibial stress and strain variables estimated using 2D beam theory and 3D finite element analysis across foot strike conditions (mean (SD)).

	**hRFS**	**iRFS**	**iFFS**	**Main effect**	**Effect size**	***Post-hoc***
					**(Kendall’s W)**	
**2D beam theory**						
Peak anterior tensile stress (MPa)	76.8	64.2	89.8	*p* < 0.001	0.762	A, B, C
	(26.2)	(20.0)	(31.2)			
**3D finite element analysis**						
95th percentile PMvM strain (*μɛ*)	3,625.9	3,067.4	4,260.0	*p* < 0.001	0.670	A, B, C
	(955.7)	(709.4)	(1,222.3)			
Strained volume >3,000µ*ɛ* (mm^3^)	5,916.0	3,071.3	8,716.5	*p* < 0.001	0.670	A, B, C
	(4,333.0)	(2,723.6)	(4,397.6)			
Peak principal tensile stress (MPa)	82.9	69.1	98.4	*p* < 0.001	0.704	A, B, C
	(21.9)	(15.4)	(27.4)			
Peak principal compressive stress (MPa)	−98.4	−81.4	−114.3	*p* < 0.001	0.799	A, B, C
	(26.5)	(20.5)	(32.4)			

**Notes.**

Effect sizes are reported as Kendall’s *W* for Friedman tests.

Significant *post-hoc* pairwise differences (*p*_corr_ < 0.05) are indicated as follows:

A = habitual rearfoot strike (hRFS) significantly differs from imposed rearfoot strike (iRFS).

B = habitual rearfoot strike (hRFS) significantly differs from imposed forefoot strike (iFFS).

C = imposed rearfoot strike (iRFS) significantly differs from imposed forefoot strike (iFFS). PMvM: pressure-modified von Mises.

#### Exploratory sex-based comparisons

**Figure 3:**
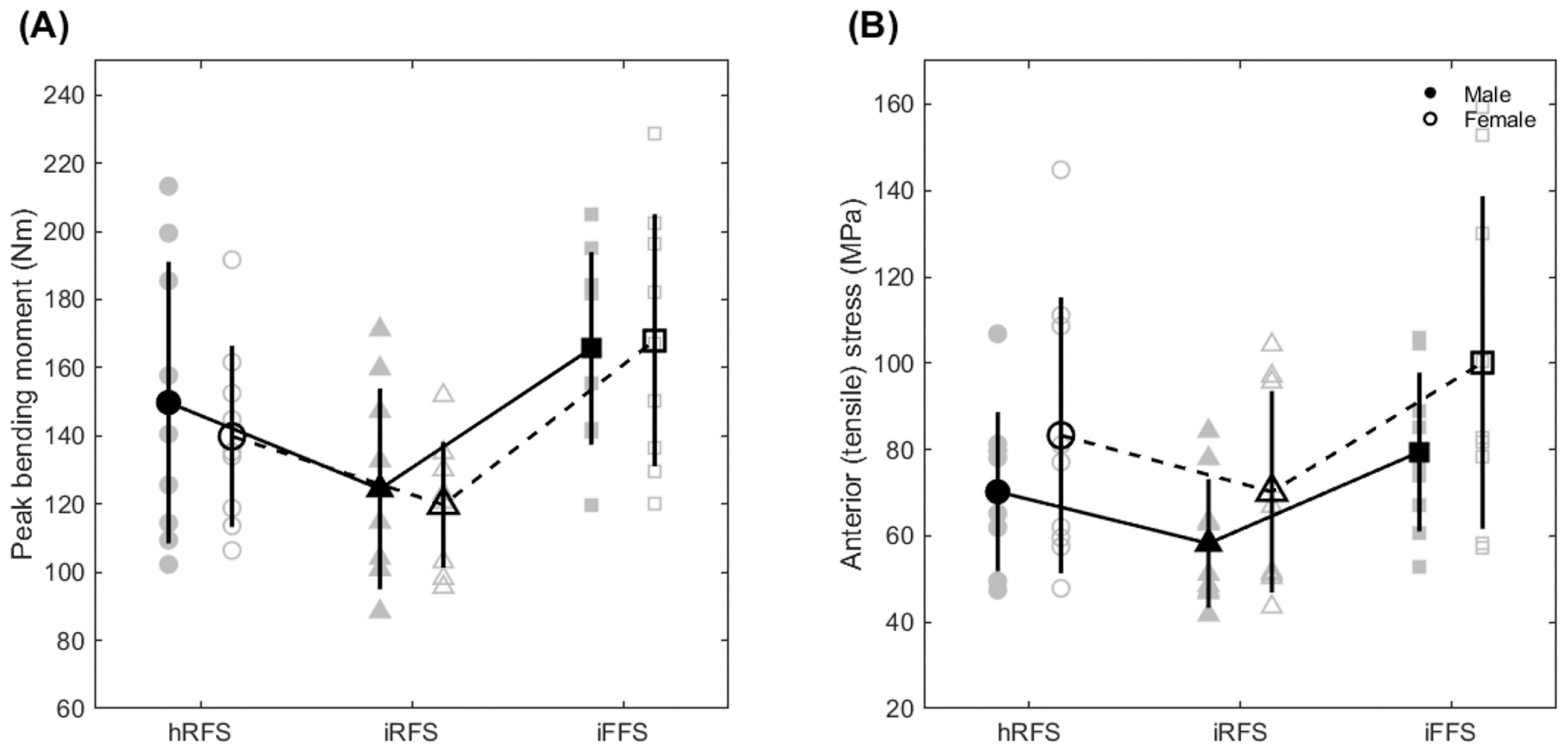
Comparison of peak tibial bending moment between males (*n* = 9) and females (*n* = 9) (A), and anterior tensile stress (B) across foot strike conditions. Values were estimated using a 2D beam theory–based model at the distal 1/3 of the tibia. Gray markers indicate individual participants. Black filled markers and solid lines represent males; open markers and dashed lines represent females. Error bars show ±1 standard deviation. hRFS, habitual rearfoot strike; iRFS, imposed rearfoot strike; iFFS, imposed forefoot strike.

Exploratory descriptive statistics for sex-based differences in distal-third tibial CSA and peak anterior tibial stress are presented in Table 4. Figure 3 illustrates sex-based differences in peak tibial bending moment and anterior tensile stress across foot strike conditions, with all variables estimated at the distal third of the tibia using the 2D beam theory approach. No formal statistical testing was performed, and these comparisons are intended to provide contextual information regarding sex-related variability.

**Table 4:** Exploratory descriptive statistics for males and females in distal third tibial cross-sectional area (CSA) and peak anterior tibial stress across foot strike conditions, estimated using 2D beam theory. Data are presented as mean (SD). hRFS, habitual rearfoot strike; iRFS, imposed rearfoot strike; iFFS, imposed forefoot strike.

	**Male**	**Female**
CSA (mm^2^)	419.2	366.9
	(76.2)	(41.8)
**Anterior stress (MPa)**		
hRFS	70.2	83.3
	(18.4)	(32.0)
iRFS	58.2	70.2
	(14.9)	(23.3)
iFFS	79.4	100.2
	(18.4)	(38.5)

### 3D finite element analysis

**Figure 4:**
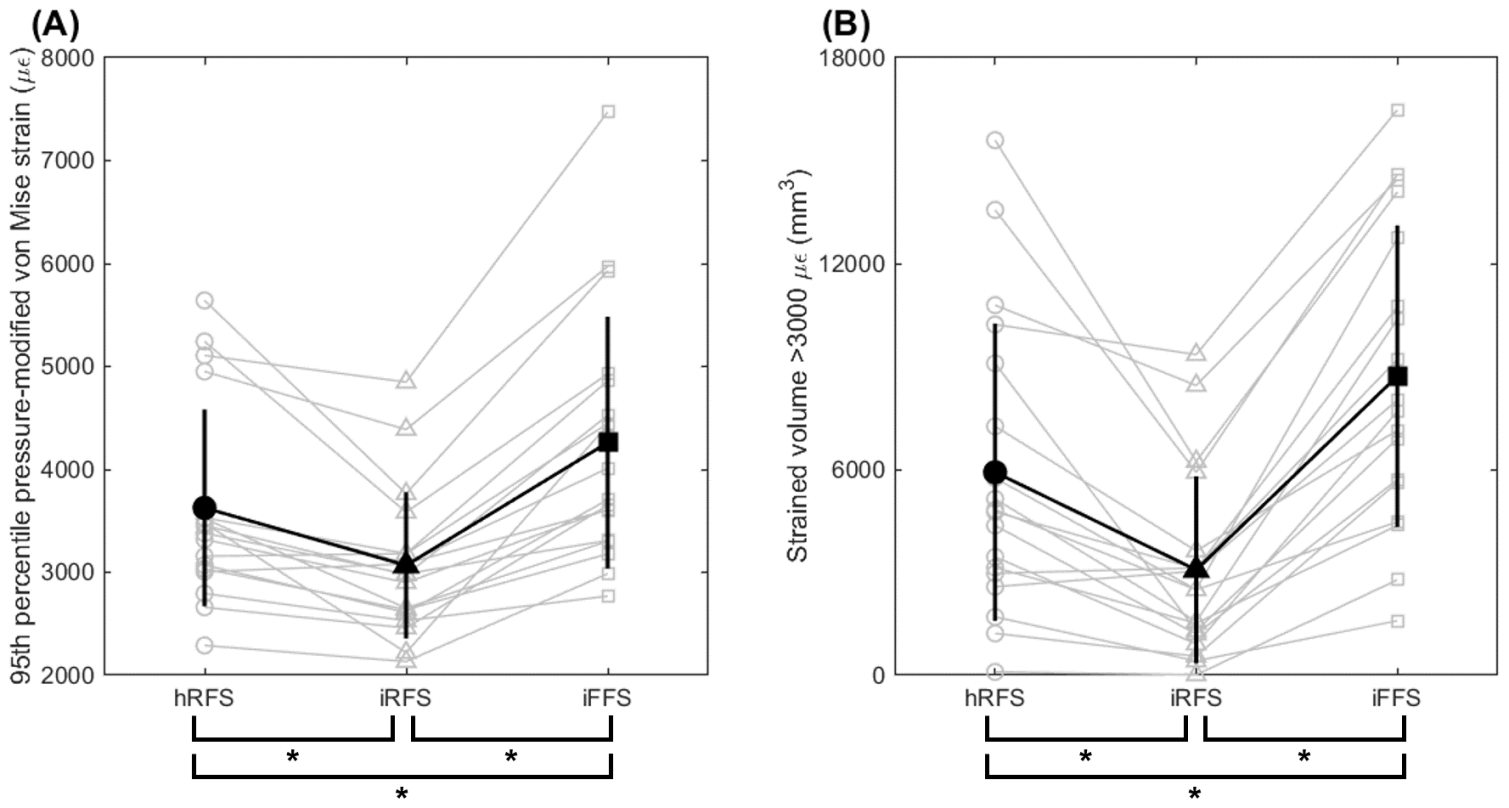
Strain-related outcomes estimated using 3D finite element analysis (FEA) across foot strike conditions. The 95th percentile pressure-modified von Mises strain (µ*ϵ*) (A) and strained volume above 3000 µ*ϵ* (mm^3^) (B) are shown. Individual participant values are shown with condition-specific markers and connected by light gray lines to illustrate within-participant trends. Black filled markers and vertical error bars indicate the mean ± SD. hRFS, habitual rearfoot strike; iRFS, imposed rearfoot strike; iFFS, imposed forefoot strike. An asterisk (*) indicates a significant *post-hoc* difference between the connected conditions (*p*_corr_ < 0.05).

Figures 2C and 2D illustrate peak principal tensile and compressive stresses across foot strike conditions, with individual participant responses shown across conditions. Figure 4 presents strain-related outcomes estimated using FEA, highlighting within-participant changes across foot strike conditions.

#### 95th pressure-modified von Mises strain

**Figure 5:**
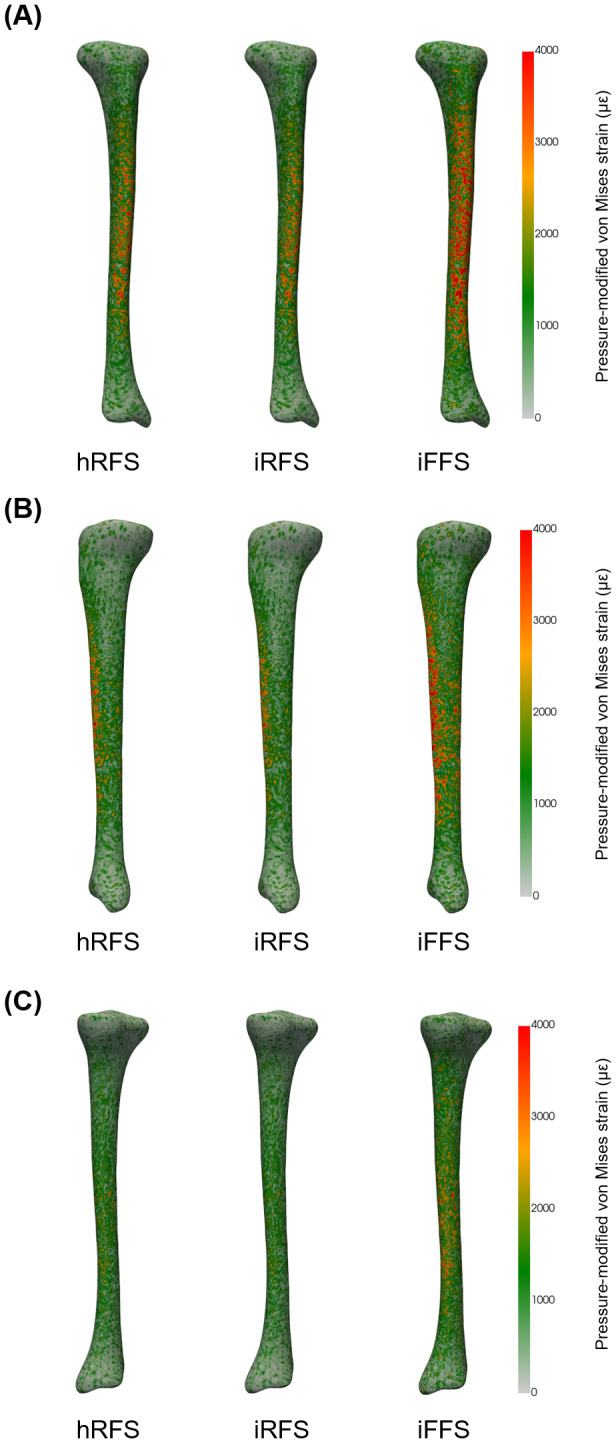
Anterior (A), lateral (B), and posterior (C) views of pressure-modified von Mises equivalent strain (µ*ϵ*) distribution in the tibia during running under three foot strike conditions. A representative participant was chosen for visualization. hRFS, habitual rearfoot strike; iRFS, imposed rearfoot strike; iFFS, imposed forefoot strike.

Figure 5 illustrates the distribution of pressure-modified von Mises equivalent strains for each foot strike condition. There was a main effect of foot strike pattern on the 95th percentile pressure-modified von Mises strain within the tibia (*p* < 0.001, Table 3). *Post hoc* analyses revealed that an imposed forefoot strike increased the 95th percentile strain by 17.5% compared to a habitual rearfoot strike (*p*_corr_ < 0.05), and by 38.9% compared to an imposed rearfoot strike (*p*_corr_ < 0.05). Additionally, the imposed rearfoot strike showed a 15.4% lower 95th percentile strain compared to a habitual rearfoot strike (*p*_corr_ < 0.05).

#### Tibial strained volume

There was a main effect of foot strike pattern on strained volume (*p* < 0.001, Table 3). *Post hoc* analyses revealed that running with an imposed forefoot strike increased strained volume by 47.3% compared to a habitual rearfoot strike (*p*_corr_ < 0.05), and by 183.8% compared to an imposed rearfoot strike (*p*_corr_ < 0.05). Additionally, strained volume was 48.1% lower during an imposed rearfoot strike compared to a habitual rearfoot strike (*p*_corr_ < 0.05).

#### Peak principal tensile stress

**Figure 6:**
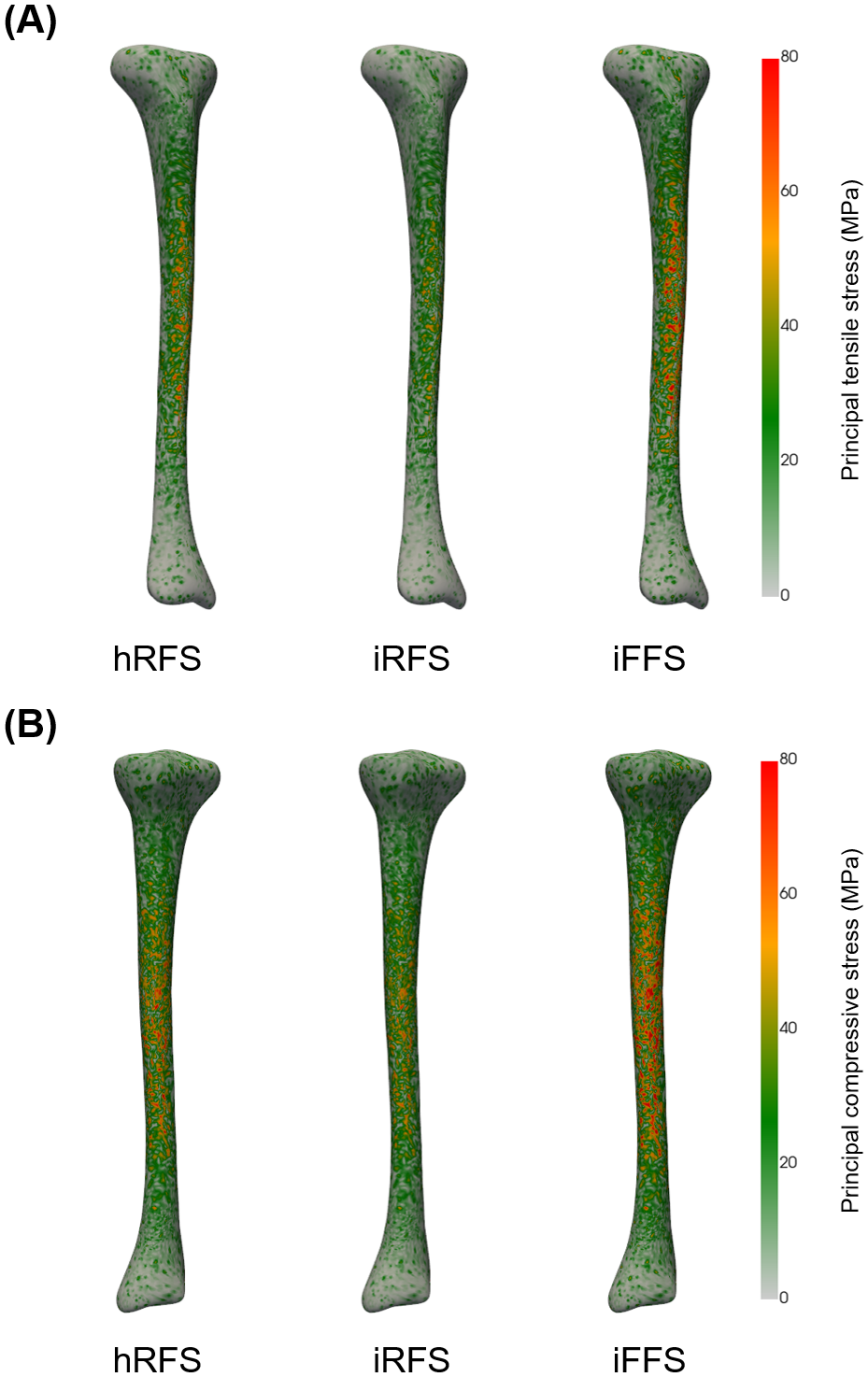
Distribution of principal tensile (A) and compressive (B) stresses (MPa) in the tibia during running under three foot strike conditions. A representative participant was chosen for visualization. hRFS, habitual rearfoot strike; iRFS, imposed rearfoot strike; iFFS, imposed forefoot strike.

There was a main effect of foot strike pattern on peak principal tensile stress (*p* < 0.001, Table 3). *Post hoc* analyses indicated that an imposed forefoot strike increased peak principal tensile stress by 18.8% compared to a habitual rearfoot strike (*p*_corr_ < 0.05), and by 42.3% compared to an imposed rearfoot strike (*p*_corr_ < 0.05, Fig. 6A). Additionally, an imposed rearfoot strike resulted in a 16.5% lower peak principal tensile stress than a habitual rearfoot strike (*p*_corr_ < 0.05).

#### Peak principal compressive stress

There was a main effect of foot strike pattern on peak principal compressive stress (*p* < 0.001, Table 3). *Post hoc* analyses revealed that an imposed forefoot strike increased peak principal compressive stress by 16.1% compared to a habitual rearfoot strike (*p*_corr_ < 0.05), and by 35.8% compared to an imposed rearfoot strike (*p*_corr_ < 0.05, Fig. 6B). Additionally, an imposed rearfoot strike resulted in a 14.5% lower peak principal compressive stress compared to a habitual rearfoot strike (*p*_corr_ < 0.05).

## Discussion

This study aimed to quantify the reconstruction errors of participant-specific tibial geometry using a simplified 4-marker SSM approach, and to compare tibial loading estimates when running with different foot strike patterns using two modeling methods (2D beam theory and 3D finite element analysis). It was hypothesized that the 4-marker configuration would yield greater reconstruction errors than the 9-marker configuration, and that reconstruction accuracy would differ according to the level of PCA constraint applied. Additionally, it was hypothesized that the proposed modeling framework would robustly capture consistent tibial loading trends across different foot strike patterns using both modeling approaches.

The simplified 4-marker set (±2 SD) exhibited statistically lower reconstruction accuracy compared to the established 9-marker set (±3 SD), resulting in a 17.2% difference in the Jaccard index. The Jaccard index values for the 4-marker configurations ranged from 0.49 to 0.55, indicating that approximately half of the reconstructed tibial volume overlapped with the MRI-based reference geometry. Mean surface errors of 8.7–10.1 mm further reflect the magnitude of absolute geometric deviation and should be considered when interpreting individual-level accuracy. This finding aligns with previous research by Bruce et al. (2022b), who reported improved reconstruction accuracy with a higher number of anatomical landmarks (14 *vs.* 9 landmarks). Specifically, Bruce et al. (2022b) showed that reconstructions based on nine palpable landmarks reduced root mean square error by 9–15% and maximum surface error by 12–17% compared to isometric scaling. Using 14 landmarks resulted in additional but relatively modest accuracy improvements compared to the nine landmarks. Although our 4-marker configuration was less accurate than the 9-marker configuration, the chosen landmarks (medial femoral condyle, lateral femoral condyle, medial malleolus, and lateral malleolus) are standard anatomical reference points commonly employed in clinical and laboratory practice, making them highly practical for biomechanical research. Within the 4-marker configurations, the ±1 SD constraint produced slightly higher Jaccard values (0.55 *vs.* 0.53) and lower mean surface errors (8.70 mm *vs.* 8.79 mm) than the ±2 SD constraint. However, the ±2 SD configuration was selected to permit greater shape variation within the PCA bounds while maintaining similar reconstruction accuracy to the ±1 SD condition, thereby allowing improved representation of participant-specific anatomical variability. The ±3 SD constraint resulted in lower Jaccard values (0.49) and higher surface errors and was therefore not used for participant-specific modeling.

Tibial lengths reconstructed using the 4-marker set (±2 SD) demonstrated a significant positive correlation with lengths measured directly from motion capture markers (Table 2), with an average descriptive difference of 1.3% between methods. Keast, Bonacci & Fox (2023) reported that PC 1, which accounts for 84.2% of tibia–fibula shape variation, predominantly captures general size-related variation, including tibial length and width, likely explaining the observed correlation. Accurate reconstruction of tibial length is important for biomechanical modeling, as it directly influences the moment arm of ankle joint contact forces and thus affects the magnitude of internal tibial loading. Even small differences in reconstructed length may influence joint moment arms and internal loading estimates, and this should be considered when interpreting model outputs.

The tibial CSA at the distal third reconstructed using the 4-marker configuration (393.1 ± 65.4 mm^2^) was comparable to values previously reported in CT and X-ray based studies using hollow elliptical modeling, including CT derived estimates of 420.7 ± 64.4 mm^2^ (Derrick et al., 2016) and X-ray based estimates of 370.9–389.7 mm^2^ (Meardon et al., 2015). These similarities are likely attributable to the fact that the participants in the present study, the reference population used to develop the SSM (Keast, Bonacci & Fox, 2023), and those included in previous imaging studies, were all young healthy adults. The SSM approach using a simplified 4-marker set provides a practical alternative to imaging-based acquisition of participant-specific geometry, which can then be used for finite element analysis (Baggaley et al., 2024). However, the absolute geometric errors introduced may limit its suitability for precise individual assessments or subtle between-group comparisons. Nevertheless, as demonstrated by Khassetarash et al. (2023), it remains highly suitable for evaluating relative differences or general trends across experimental conditions, where absolute accuracy is less critical.

**Figure 7:**
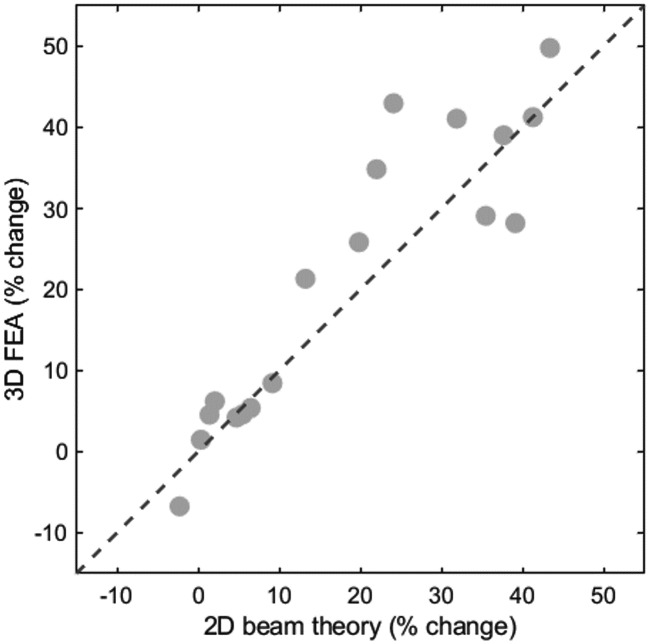
Comparison of participant-specific percentage changes in peak anterior tensile stress between 2D beam theory and 3D finite element analysis (FEA) for the transition from habitual rearfoot strike (hRFS) to imposed forefoot strike (iFFS). Each marker represents one participant. The dashed line represents the line of identity.

In the present study, tibial stress calculated using simplified 2D beam theory and detailed 3D finite element analysis showed consistent directional trends. Accordingly, this comparison was intended to be qualitative and trend-based, rather than a direct quantitative comparison. These findings suggest that the two modeling approaches should be viewed as complementary, with the choice of method depending on the specific research context and objectives. Individual participant trends were consistent across modeling approaches, as illustrated by the within-participant trajectories shown in Fig. 2 (stress) and Fig. 4 (strain). To further quantify within-participant agreement, percentage changes in peak anterior tensile stress between hRFS and iFFS were calculated for each participant using both approaches. All participants demonstrated concordant directional changes across methods. The mean percentage increase was 18.7% for the 2D model and 21.2% for the 3D model. The mean absolute difference in percentage change between methods was 5.3 ± 5.1%. Similar directional consistency was observed for compressive stress comparisons (Fig. 7).

At the group level, peak anterior tensile stress increased by 17.0% in the 2D model and 18.8% in the 3D model when participants changed from a rearfoot strike to a forefoot strike. Peak anterior tensile stress under habitual rearfoot striking was 76.8 MPa (2D) and 82.9 MPa (3D)(Table 3). This similarity, despite fundamental differences between the models, likely reflects the use of consistent experimental conditions, including identical participants and loading inputs (ankle joint reaction forces and 11 muscular forces). Baggaley et al. (2024) argued that simplified 2D models may not fully represent the 3D geometry of the tibia and may misrepresent stress distributions. The 3D finite element analysis provides more detailed information, including stress and strain distributions across the bone surface. Nonetheless, qualitative comparisons with other finite element studies suggest that peak stresses and strains occur in broadly consistent anatomical regions during running (Fig. 6). Thus, for typical healthy bone geometries, and particularly in within-participant study designs where tibial geometry remains constant across conditions, detailed 3D finite element analysis may not be necessary to detect relative, condition-dependent changes in tibial loading. Previous work has shown that simplified beam theory may underestimate axial stress and overestimate bending stress relative to finite element analysis, particularly in comparisons involving substantial inter-individual geometric variability (Brassey et al., 2013). However, under the present within-participant design, simplified beam theory appears adequate for capturing relative changes despite its limitations in absolute stress estimation.

Building on the trend-based agreement observed between the 2D and 3D approaches, the 3D finite element analysis exhibited general findings consistent with the 2D beam theory approach, indicating higher strained volumes and 95th percentile pressure-modified von Mises strains under an imposed forefoot strike compared to a habitual and an imposed rearfoot strike. Specifically, strained volume was 47.3% and 183.8% greater, and 95th percentile strain was 17.5% and 38.9% greater, respectively. In the present study, strained volume ranged from approximately 3071 mm^3^ (imposed rearfoot strike) to 8717 mm^3^ (imposed forefoot strike). Previous running studies also reported wide ranges of strained volumes, *e.g.*, 6,000–10,000 mm^3^ (Khassetarash, Nigg & Edwards, 2024) and 500–2500 mm^3^ (Baggaley et al., 2024). This variability in strained volume magnitudes has been attributed by Baggaley et al. (2024) to individual differences in bone geometry and running biomechanics. Additionally, differences between overground running (current study) and treadmill running conditions employed in prior studies (Baggaley et al., 2024; Khassetarash, Nigg & Edwards, 2024) may further account for differences in observed tibial loading outcomes. Differences in modeling assumptions, such as boundary conditions, material property definitions, and loading application methods, may also contribute to these discrepancies. Therefore, the primary focus of this study was the relative change in tibial loading across foot strike conditions rather than absolute values, given their sensitivity to modeling assumptions.

Our findings regarding the 95th pressure-modified von Mises strains differ from the results of Chen et al. (2016), who reported no significant differences in peak principal strains across foot strike patterns at a lower running speed (2.5 m ⋅ s^−1^). These differences likely reflect variations in running speed or other experimental conditions, as differences between foot strike conditions in the present study were also detected using the simplified 2D beam theory. Nonetheless, methodological differences may also contribute, as Chen et al. (2016) analyzed peak principal strains, while the current study utilized the pressure-modified von Mises strain—a multiaxial strain criterion validated for quasi-brittle materials exhibiting asymmetric strength characteristics under compression and tension (De Vree, Brekelmans & van Gils, 1995; Haider et al., 2021).

Consistent with our previous findings derived from the same running dataset without participant-specific tibial geometry (Han et al., 2025), an imposed forefoot strike increased tibial bending moment in the sagittal plane, primarily due to increased mechanical demands placed on the plantar flexors, as reflected by the greater muscular contribution to the bending moment and higher plantar flexor forces reported in that study. Although the current sample size (nine males, nine females) precluded formal sex-based statistical inference, exploratory descriptive comparisons were included to examine whether the proposed modeling framework remains robust to known sex-related variability in tibial geometry (Table 4). The SSM-based reconstructions yielded larger mean cross-sectional areas at the distal third of the tibia in males than females. While tibial bending moments, which were not normalized to bone geometry, showed comparable magnitudes between sexes (Fig. 3A), anterior tensile stress exhibited clearer sex-related differences (Fig. 3B), consistent with underlying geometric variation. These observations suggest that the proposed framework preserves biologically meaningful variability in bone geometry rather than obscuring it, even in the absence of participant-specific medical imaging. However, more accurate geometric reconstructions would likely be required to confidently detect differences between independent participant groups. These findings do not contradict prior review work discussing potential benefits of forefoot striking under specific conditions (Davis, Rice & Wearing, 2017). That review primarily considered barefoot or minimal-footwear contexts and the potential effects of longer-term adaptation. In contrast, the present study examined the acute mechanical response to an imposed forefoot strike in habitual rearfoot runners wearing conventional footwear, where increased plantar-flexor demand provides a plausible explanation for the observed increase in tibial loading.

This study has several limitations that should be considered. Although the use of a simplified 4-marker set SSM was a primary focus of the investigation, neither the models used in this study nor those reported in the literature have been validated against injury outcomes. This absence of outcome-level validation limits the ability to assess the clinical relevance of the predicted tibial stresses and strains. Additionally, the SSM approach employed in this study could be less accurate when applied to populations with substantially different characteristics from those in the present study or those used to develop the SSM of the tibia-fibular (Keast, Bonacci & Fox, 2023), such as older or younger individuals, due to differences in tibial morphology. Therefore, caution is warranted in generalizing these findings beyond the young and healthy adult population from which the model was developed. In addition, although MRI-derived geometries were used as reference data to evaluate reconstruction accuracy, the absence of participant-specific CT imaging precludes validation against a definitive imaging-based reference. Muscular forces were estimated using static optimization, which involves simplifying assumptions and does not capture individual differences in muscle strength or activation capacity. Furthermore, the running protocol included only habitual rearfoot strikers, potentially limiting the generalizability of the findings. The 10 m runway may also not have fully ensured steady-state running throughout the capture volume. These protocol-related limitations have been discussed in detail in previous work (Han et al., 2025). Therefore, the increased tibial loading observed during forefoot striking should be interpreted as an acute response, rather than a long-term biomechanical adaptation.

## Conclusion

Participant-specific tibial geometries generated from only four anatomical shank markers exhibited a 17.1% lower geometric overlap (Jaccard index) compared with an established 9-marker approach, while reconstructed tibial length demonstrated a significant positive correlation with direct marker-based measurements, with a small average difference of 1.3% between methods. The relative increase in anterior tensile tibial stress during imposed forefoot striking compared with habitual rearfoot striking was 17.0% and 18.8%, when quantified using a two-dimensional beam theory approach and a three-dimensional finite element approach, respectively. Participant-specific percentage changes were directionally consistent across methods, with a mean absolute between-method difference of 5.3%. Given the consistent directional trends in relative tibial loading observed between the simplified two-dimensional model and the computationally intensive three-dimensional finite element analysis, the combination of 4-marker statistical shape model–derived geometry and two-dimensional beam theory may provide a practical alternative for estimating within-participant or within-group changes in tibial loading in healthy runners using Keast et al.’s open-source dataset (Keast, Bonacci & Fox, 2023) when medical imaging is unavailable.

## Supplemental Information

10.7717/peerj.21328/supp-1 Supplemental Information 1 Supplementary Material Detailed descriptions of all variables included in the ankle joint contact force dataset, including units, normalization procedures, and condition labels.

10.7717/peerj.21328/supp-2 Supplemental Information 2 Ankle joint contact force dataset used for tibial loading analysis Ankle joint contact force components (axial, anterior–posterior, and medial–lateral) for 18 participants under three foot strike conditions: habitual rearfoot, imposed rearfoot, and imposed forefoot strike conditions. Values correspond to the time of peak resultant force and are provided in both raw (*N*) and body-weight normalized formats. Data were scaled to 90% of the total force to account for fibula exclusion and are provided in both raw (*N*) and body-weight normalized form in a MATLAB (.mat) table.

## Additional Information and Declarations

### Data Availability

The following information was supplied regarding data availability: The data is available in the Supplemental File.
